# Editorial: Biological rotary nanomotors

**DOI:** 10.3389/fmicb.2022.1012681

**Published:** 2022-09-21

**Authors:** Michael D. Manson, Beiyan Nan, Pushkar P. Lele, Jun Liu, Thomas M. Duncan

**Affiliations:** ^1^Department of Biology, Texas A&M University, College Station, TX, United States; ^2^Artie McFerrin Department of Chemical Engineering, Texas A&M University, College Station, TX, United States; ^3^Microbial Sciences Institute, Department of Microbial Pathogenesis, Yale University, New Haven, CT, United States; ^4^Department of Biochemistry & Molecular Biology, Upstate Medical University, Syracuse, NY, United States

**Keywords:** rotary nanomotors, flagella, archaella, gliding, outer membrane transport, ATP synthase

This Research Topic of 20 review articles covers the incredible variety of molecular rotary nanomachines that have evolved over billions of years. The details of their molecular mechanisms are well-known for the Bacterial Flagellar Motor (BFM) and FoF1 ATP synthase/ATPase. They are inferred by analogy to the BFM for others: the 5:2 motors of gliding bacteria and the 5:2 motors of the Ton and Tol systems that govern events at the outer membrane of gram-negative bacteria. All of these systems couple a transmembrane ion motive force to useful work. Two additional articles consider the unique propulsion generator of the Archaea, the archaellum, which appears to be driven entirely by ATP hydrolysis and has an evolutionary history completely independent of those for the 5:2 motors and FoF1.

This Research Topic originated with Mike Manson, who was inspired by the recently published structures of 5:2 rotary motors and the desire to create a fitting tribute to Howard Berg, who introduced rotary motors into biology. The effort was augmented by recruitment of Tom Duncan, Pushkar Lele, Jun Liu, and Beiyan Nan, who possess the expertise to select the authors best able to tell the story of rotary nanomotors and to edit their contributions.

The Research Topic has six sections. The first two chapters serve as an introduction to the history, structure, and energetics of rotary motors driven by an ion motive force. The second of these provides a detailed consideration of the structure of 5:2 rotary motors and how their function and energetics compare with the Fo motor of ATP synthases, which has a gearing ratio ranging from 8:1 to 15:1 in different species. The next seven articles provide detailed reviews about different aspects and incarnations of BFMs. The following two articles discuss the function of ion-powered motors in gliding bacteria, which do not depend on flagella. Next, two reviews cover the Exb/Ton and Tol/Pal systems, both of which are driven by 5:2-type motors. As a segue into the FoF1 motors that drive ATP synthesis, the next two articles cover the archaellum, a helical propeller like the bacterial flagellum, but one driven by ATP hydrolysis rather than an ion motive force and possessing a completely different evolutionary trajectory. The last five articles review different aspects of what is known about the FoF1 ATP synthase, a crucial and ubiquitous energy-transducing enzyme that couples two rotary motor devices.

The editors thank all 49 authors who generously agreed to put their collective knowledge about rotary nanomotors into words. They represent the leading research groups worldwide studying rotary molecular machines. The positive responses to our invitations were doubtless aided by the recognition that this was a tribute to the visionary insight of Howard Berg, whose paper with Robert Anderson in 1973 first declared that “Bacteria Swim by Rotating Helical Filaments.”

We hoped that Howard would write an introduction to this Research Topic. Unfortunately, he passed away on December 30, 2021, just as it became clear that our vision was going to become reality. That sad event adds both poignancy and timeliness to this Research Topic. We also want to thank the four editors who supplemented the efforts of the five designated guest editors mentioned above and the 33 reviewers who, together with the guest editors, ensured that everything presented in the reviews was as clear, complete, and accurate as possible.

We hope this Research Topic serves as a fitting tribute to Howard Berg, the unconventional intellect whose insight ushered in an entire field of molecular biophysics: the study of rotary molecular motors. Savor what is presented here and anticipate the new revelations which doubtless await in the coming years.

## Author contributions

All authors listed have made a substantial, direct, and intellectual contribution to the work and approved it for publication.

## Conflict of interest

The authors declare that the research was conducted in the absence of any commercial or financial relationships that could be construed as a potential conflict of interest.

## Publisher's note

All claims expressed in this article are solely those of the authors and do not necessarily represent those of their affiliated organizations, or those of the publisher, the editors and the reviewers. Any product that may be evaluated in this article, or claim that may be made by its manufacturer, is not guaranteed or endorsed by the publisher.

